# Sex hormone-binding globulin provides a novel entry pathway for estradiol and influences subsequent signaling in lymphocytes via membrane receptor

**DOI:** 10.1038/s41598-018-36882-3

**Published:** 2019-01-09

**Authors:** Andrea Balogh, Eva Karpati, Andrea E. Schneider, Szabolcs Hetey, Andras Szilagyi, Kata Juhasz, Gloria Laszlo, Petronella Hupuczi, Peter Zavodszky, Zoltan Papp, Janos Matko, Nandor Gabor Than

**Affiliations:** 1 Department of Immunology, Eotvos Lorand University, Budapest, Hungary; 2 Systems Biology of Reproduction Lendulet Research Group, Institute of Enzymology, Research Centre for Natural Sciences, Hungarian Academy of Sciences, Budapest, Hungary; 3 Laboratory of Structural Biophysics, Institute of Enzymology, Research Centre for Natural Sciences, Hungarian Academy of Sciences, Budapest, Hungary; 4 Maternity Private Department, Kutvolgyi Clinical Block, Semmelweis University, Budapest, Hungary; 5 First Department of Pathology and Experimental Cancer Research, Semmelweis University, Budapest, Hungary

## Abstract

The complex effects of estradiol on non-reproductive tissues/cells, including lymphoid tissues and immunocytes, have increasingly been explored. However, the role of sex hormone binding globulin (SHBG) in the regulation of these genomic and non-genomic actions of estradiol is controversial. Moreover, the expression of SHBG and its internalization by potential receptors, as well as the influence of SHBG on estradiol uptake and signaling in lymphocytes has remained unexplored. Here, we found that human and mouse T cells expressed SHBG intrinsically. In addition, B lymphoid cell lines as well as both primary B and T lymphocytes bound and internalized external SHBG, and the amount of plasma membrane-bound SHBG decreased in B cells of pregnant compared to non-pregnant women. As potential mediators of this process, SHBG receptor candidates expressed by lymphocytes were identified *in silico*, including estrogen receptor (ER) alpha. Furthermore, cell surface-bound SHBG was detected in close proximity to membrane ERs while highly colocalizing with lipid rafts. The SHBG-membrane ER interaction was found functional since SHBG promoted estradiol uptake by lymphocytes and subsequently influenced Erk1/2 phosphorylation. In conclusion, the SHBG-SHBG receptor-membrane ER complex participates in the rapid estradiol signaling in lymphocytes, and this pathway may be altered in B cells in pregnant women.

## Introduction

17β-estradiol (E2) was first known as a sex steroid primarily acting on reproductive tissues in vertebrates^1,2^. Since then an increasing body of evidence has revealed that E2 is capable of influencing and regulating the development, growth, differentiation, and function of non-reproductive tissues, as well^3,4^. Cells of the innate and adaptive immune systems are both influenced by E2^5–7^. Therefore, it is not surprising that E2 strongly impacts immune functions and the incidence of autoimmune diseases in non-pregnant and pregnant women^4,8–10^.

Similar to its effects, the mode of E2 action is also complex and can be divided into classical (genomic) and non-classical (non-genomic) pathways^11^. The effect of E2 through the classical mechanism involves interaction with its intracellular receptors, estrogen receptor alpha (ERα) and beta (ERβ)^12,13^. These receptors are encoded by separate genes (*ESR1* and *ESR2*) and have numerous mRNA splice variants. Three ERα isoforms (with molecular weights of 66, 46 and 36 kDa), and several ERβ isoforms have been identified^14–16^. ERα, ERβ, and their isoforms are expressed by most immune cells, although at various expression levels^17,18^. After ligand binding, homo- or heterodimers of ERα and ERβ are formed and bound to estrogen responsive elements (EREs) in the genome^19,20^. Additionally, ERs may indirectly bind to DNA *via* their interaction with other transcription factors, such as nuclear factor kappa B^21^. Since this mechanism involves nuclear translocation of ERs and target gene transcription or repression, the onset of the effect is fairly slow (hours, days).

In contrast, non-genomic E2 signaling, including calcium mobilization and phosphorylation of extracellular signal-regulated kinase (Erk) and protein kinase B (PKB also known as Akt), takes place within seconds to minutes^22^. These rapid actions of E2 are mediated by membrane estrogen receptors (mERs)^23^, which mainly originate from classical ERs by various modifications. Palmitoylation of the 66 kDa ERα and the truncated ERα splice variants enable their insertion into the plasma membrane^8,24^; association of ERβ with plasma membrane caveola components has also been reported^23^. In addition, G-protein coupled ER (GPER also known as GPR30) may also belong to the mER group^9,25^. Of importance, the existence of crosstalk between signaling pathways mediated by these receptors was also demonstrated^26,27^.

Several studies, using membrane-impermeable E2-BSA conjugate as a mER ligand, confirmed that mERs with an extracellular binding site may exist and mediate signals in the majority of immune cells^22,28,29^. A recent *in vitro* model pointed out that at least six forms of ERs with various subcellular localization may be present in mouse lymphocytes to mediate rapid signaling, depending on their actual localization^30^. Moreover, their localization may be mutually affected by the fluctuating E2 level. However, many questions still have remained open regarding the complexity and fine-regulation of immune cells by E2^31^.

The overall view, however, is further complicated when taking into account E2-binding transport proteins and their specific receptors involved in the internalization and signaling of E2^32,33^. A widely known protein that binds E2 is the sex hormone binding globulin (SHBG)^34^. It is produced primarily by the liver, but its expression was also detected in many sex steroid-responsive tissues, such as the placenta, testis or brain^35–37^. Functional SHBG is a Ca^2+^-promoted dimer, which may bind two estrogen ligands with an affinity of four to five orders of magnitude higher than that of albumin^38,39^. Of note, approximately 38% of E2 is bound to SHBG, while 60% is bound to albumin, and only 2% is considered to be free in the circulation of women in the follicular phase^40^. SHBG is generally known as a carrier protein that keeps its ligands physically separated from the environment; thus, controlling the amount of free E2 for target cells^33,41^, as formulated by the “free hormone hypothesis”. Nevertheless, the free hormone hypothesis is not likely to be valid for all hormones with respect to all tissues^42,43^. In accordance with this statement, it has been shown that SHBG is internalized by e.g. neurons or prostate cancer cells alone or in complex with sex steroids^44,45^. However, the expression of SHBG and its internalization by potential SHBG receptors (R_SHBG_), such as the low density lipoprotein receptor-related protein-2 (*LRP2*) encoding megalin^6^, in lymphocytes has not yet been investigated. Furthermore, it is also not known whether SHBG can influence E2 uptake and subsequent signaling events that regulate immune cell functions in infections, autoimmunity or pregnancy. Of note, SHBG, was identified as a predictive biomarker of asymptomatic spontaneous preterm delivery in a recent ‘omics’ study^46^, underlining the importance of this protein during gestation.

Therefore, in the present study we examined the intrinsic expression of SHBG in lymphocytes as well as its binding and internalization into these cells. We also investigated the amount of surface-bound SHBG on peripheral blood B and T lymphocytes in pregnancy. We searched for R_SHBG_ as well and explored its plasma membrane compartmentalization. Finally, we investigated the effect of SHBG on E2 uptake and E2-induced rapid signaling in lymphocytes. Our results suggest that the SHBG-SHBG receptor-mER complex participates in rapid mER signaling in lymphocytes. This pathway may be altered in B lymphocytes in pregnant women that may be associated with changes in maternal immune homeostasis.

## Results

### Amongst lymphocytes, SHBG is expressed by T but not B cells

**Figure 1:**
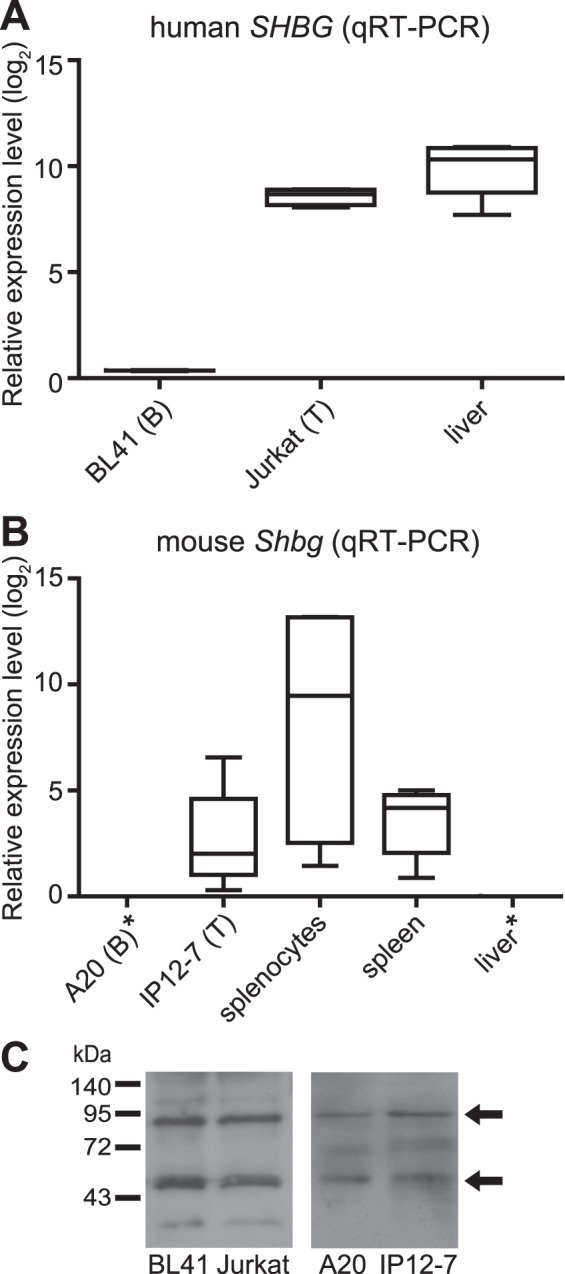
Expression of SHBG by lymphocytic cells. Box-plots (whiskers: min to max) represent *SHBG*/*Shbg* mRNA expression levels, measured by qRT-PCR, in (**A**) human B cells (BL41), T cells (Jurkat), as well as in (**B**) mouse B cells (A20), T cells (IP12-7), splenocytes, spleen. Liver served as positive control in human and negative control in mouse. Relative expression level (−ΔCt with an arbitrary zero point) is denoted on the Y-axis. Three independent qRT-PCR experiments were performed in duplicates. Asterisks denote expression under the detection limit. (**C**) Representative Western blot images show SHBG protein in the cell lysate of human (BL41, Jurkat) and mouse (A20, IP12-7) lymphocytes. The two bands represent monomeric (~45 kDa) and dimeric (~90 kDa) forms of the protein.

SHBG has an important role in the regulation of free E2 availability to various target cells, but in lymphocytes this mechanism is unexplored. Therefore, we first examined whether human and mouse B and T lymphocytes can express *SHBG* and *Shbg* respectively, by exploring its expression pattern in different tissues, primary cells and cell lines of lymphoid origin using the Genevestigator web-based analysis tool and the GTEx Project, and determining its expression level by qRT-PCR and Western blot. Publicly available microarray and RNA-Seq data showed that the primary source of *SHBG* in human is the liver. However, although with a much lower expression, *SHBG* mRNA was present in the spleen and in various lymphocyte cell lines (B cells: BL41, Daudi, Raji; T cells: Jurkat, CCRF-CEM, HUT-78) as well as in primary lymphocytes (Fig. S1A). In mouse, microarray analysis showed the highest mRNA expression of *Shbg* in fetal liver, followed by B cells and T cells. Somewhat lower *Shbg* expression was found in liver, and spleen (Fig. S1B). Supporting publicly available microarray and RNA-Seq data, we found that *SHBG*/*Shbg* mRNA is expressed in T lymphocytic cells (Jurkat, IP12-7, cells) derived from both human and mouse. In contrast, B cells produced almost (BL41 human cells) or completely (A20 mouse cells) undetectable levels of *SHBG* transcripts (Fig. 1A,B). In addition, we found that mouse splenocytes (consisting mainly of B- and T lymphocytes, and a few percentages of other immune cell populations) were also the source of *Shbg*. In accordance with published data, SHBG expression was high in human liver, while it was undetectable in mouse liver (negative control). Using Western Blot, we could detect monomeric and dimeric SHBG (approx. 45 and 90 kDa, respectively) at the protein level in both lymphocyte populations in human and mouse as well (Fig. 1C). All these results suggested that although the main source of SHBG is the liver in humans, and that mouse spleen is capable of SHBG expression, this protein could be expressed by T lymphocytes as well. Since B cells contained SHBG at protein but not at mRNA level, lymphocytes may have the competence to bind and internalize this protein.

### SHBG is bound and internalized into lymphocytes

**Figure 2:**
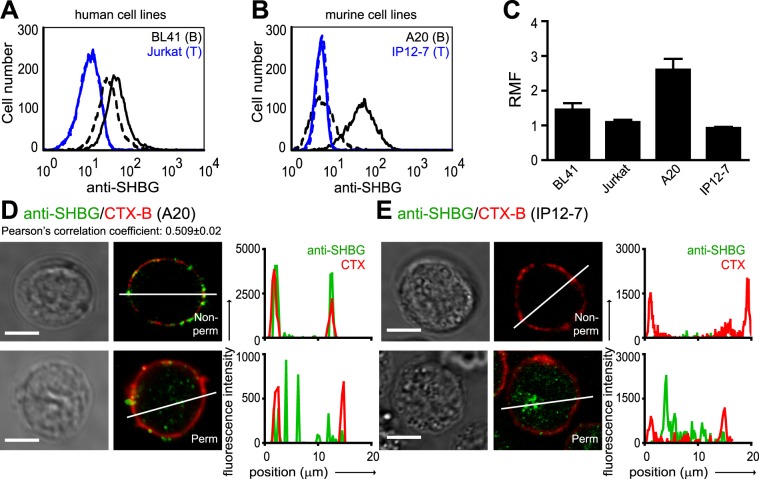
B lymphocytes can bind and internalize SHBG from the serum. (**A**,**B**) Cell surface-bound SHBG was detected on intact human and mouse B cells (BL41, A20) and T cells (Jurkat, IP12-7), using flow cytometry, by incubating the cells with anti-SHBG antibody for 20 minutes on ice, followed by A488-conjugated secondary antibody staining. Representative histograms are displayed. Continuous lines: anti-SHBG antibody; dashed lines: isotype control antibody. (**C**) The overall flow cytometric data on SHBG binding to the cell surface is represented as mean and standard error of mean (S.E.M.) values. The relative mean of fluorescence (RMF) was calculated by dividing the mean fluorescence intensity of the SHBG-specific antibody by the mean fluorescence intensity of isotype control antibody binding. (**D**) Representative confocal images (left and middle panels) show both cell membrane- (non-permeabilized cells) and intracellular (fixed and permeabilized cells) localization of SHBG (green: anti-SHBG antibody) in A20 B cells. Staining with CTX-B-A647 (red) served as visualization of the plasma membrane. Pearson’s correlation coefficient was calculated from ≥50 ROIs/sample. Line scan intensity profiles are also depicted (right panel). Scale bars: 5 µm. (**E**) Confocal images show SHBG immunostaining of IP12-7 T cells with or without permeabilization, as in D. Alexa Fluor 488, A488; Alexa Fluor 647, A647; Cholera toxin-B, CTX-B; Non-permeabilized, Non-perm; Permeabilized, Perm; region of interest, ROI.

**Figure 3:**
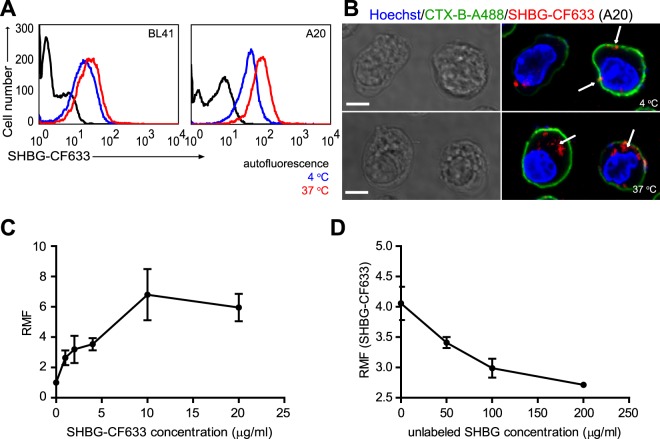
Lymphocytes can bind and internalize purified SHBG. (**A**) Binding and internalization of SHBG into BL41 and A20 B cells were detected directly using SHBG-CF633. Representative histograms are displayed. Black line: autofluorescence; blue line: incubation at 4 °C; red line: incubation at 37 °C. (**B**) Binding (upper panel) and internalization (lower panel) of SHBG-CF633 into A20 B cells were visualized by confocal microscopy. Images demonstrate mainly plasma membrane localization (white arrows) of SHBG-CF633 (red) at 4 °C (top), while its internalization (white arrows) occurred at 37 °C (bottom). CTX-B-A488 (green) and Hoechst 33342 (blue) served for counterstaining plasma membranes and nuclei, respectively. Scale bars: 5 µm. (**C**) Saturation binding curve shows specific binding of SHBG-CF633 to the surface of A20 B cells. Relative mean of fluorescence (RMF) is plotted against labeled SHBG concentration. (**D**) A20 cells were incubated with increasing amount of unlabeled SHBG for 15 minutes at 37 °C followed by the addition of SHBG-CF633. Result of competition binding assay is displayed as mean and standard error of mean (S.E.M.) values. RMF: ratio of the mean fluorescence of SHBG-CF633 and autofluorescence. Data are derived from three independent measurements. Alexa Fluor 488, A488; Cholera toxin-B, CTX-B.

Next, we investigated, whether circulating SHBG can bind to the surface of lymphocytes. Flow cytometry measurements demonstrated that SHBG was captured from the serum to surfaces of human and mouse B lymphocytes (BL41, A20, respectively) although with various extent, but not of human Jurkat and mouse IP12-7 T cells (Fig. 2A–C). Pre-incubation of mouse splenocytes or A20 B cells with purified SHBG resulted in an enhanced anti-SHBG binding, further confirming the existence of specific SHBG binding sites on surface of these cells (Fig. S2). Differential binding to lymphocytes was corroborated by confocal microscopy and subsequent line scan analysis, which clearly showed the cell membrane localization of SHBG in B cells but not in T cells (Fig. 2D,E). The high degree of colocalization between SHBG and CTX-B (Fig. 2D; colocalization index: 0.509 ± 0.02), which latter labels GM_1_/GM_3_ gangliosides in the plasma membrane, suggested that SHBG was associated mainly with lipid rafts. Taken together our qRT-PCR, flow cytometry, and confocal microscopic data, it is likely that the source of intracellular SHBG (Fig. 2D,E) in B cells is of mainly extracellular origin, while in T cells is mostly intrinsic. As a direct proof of SHBG binding and internalization, BL41 and A20 cells were incubated with fluorescent SHBG (SHBG-CF633) either at 4 °C or 37 °C, and fluorescence was measured by flow cytometry. We found that SHBG-CF633 bound to these cells both at 4 °C and 37 °C (Fig. 3A). Confocal microscopy further revealed that SHBG-CF633 remained mainly in the plasma membrane at 4 °C, while it got internalized at 37 °C (Fig. 3B). The saturating SHBG-CF633 binding (Fig. 3C) and the inhibition of its binding by increasing amount of unlabeled SHBG (Fig. 3D) corroborate the specific binding of SHBG to the cells surface of these lymphocytes.

### B cells show lower cell surface SHBG levels in pregnant than in non-pregnant women

**Figure 4:**
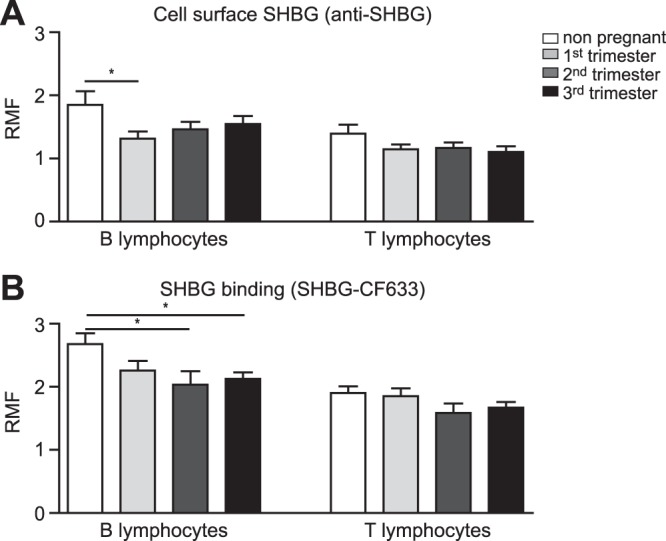
Cell surface SHBG on pregnant and non-pregnant women’s peripheral blood lymphocytes. (**A**) Cell surface-bound SHBG was detected by incubating intact human peripheral blood leukocytes with anti-SHBG antibody for 20 minutes on ice, then subsequently with an A488-conjugated secondary antibody. Cells were also stained for CD19 or CD3, in order to distinguish between B and T lymphocytes during flow cytometric measurement. The diagram shows the amount of cell surface SHBG as mean and standard error of mean (S.E.M.) values calculated from data obtained from non-pregnant women and from pregnant women in the first, second and third trimesters of pregnancy. RMF: ratio of mean fluorescence of SHBG specific antibody divided by mean fluorescence of isotype control antibody. (**B**) To detect binding of SHBG to human lymphocytes directly, isolated cells were kept in serum-free RPMI medium for 1 hour at 37 °C, then lymphocytes were incubated with SHBG-CF633 for 30 minutes at 4 °C. Population-specific antibodies were used, as described above, to discriminate B and T lymphocytes. Unpaired t-test was used for the comparison the non-pregnant group with pregnant groups (*P ≤ 0.05). Five donors were included in each group. Alexa Fluor 488, A488.

Since the amount of circulating SHBG increases in pregnancy^47^, we examined whether this is reflected in SHBG binding to lymphocyte surfaces. Therefore, we measured cell surface SHBG, reflecting the amount of plasma membrane R_SHBG_, in human primary lymphocytes obtained from non-pregnant and pregnant women from all three trimesters by flow cytometry. We detected more SHBG on the surface of B lymphocytes than in T cells both in non-pregnant and pregnant states (Fig. 4A). In contrast to what expected, SHBG staining was significantly weaker in B lymphocytes in the first trimester compared to non-pregnant healthy controls (Fig. 4A). SHBG staining tended to decrease in T cells obtained from pregnant women as well, although it did not reach statistical significance. When SHBG-CF633 was added directly, we could observe a similar binding pattern to these cell populations (Fig. 4B). Namely, (1) B cells bound more SHBG than T cells; (2) decreased SHBG binding could be detected to B cells of pregnant women compared to the non-pregnant state, reaching the statistical significance at second and third trimesters; and (3) there was a tendency of decreased SHBG binding to T cells of pregnant women compared to non-pregnant donors (Fig. 4B).

### A membrane component other than megalin is the receptor for SHBG in lymphocytes

Binding and internalization of SHBG into lymphocytes suggests that an R_SHBG_ is present in the plasma membrane of these cells. Thus, we investigated whether megalin (encoded by human *LRP2* and mouse *Lrp2*), a putative R_SHBG_^6^, can be the mediator of this binding. We explored its expression in primary lymphocytes and lymphocytes cell lines using public microarray and RNA-Seq data through Genevestigator and GTEx project, respectively, as well as own data generated by qRT-PCR. Genevestigator and GTEx data clearly demonstrated that among the investigated tissues and cells, *LRP2*/*Lrp2* transcripts were abundant in human and mouse kidneys, while spleens and lymphocytes expressed very low levels of *LRP2*/*Lrp2* (Fig. S3A,B). Our qRT-PCR results supported these findings, since neither human nor mouse lymphocytic cells expressed *LRP2* and *Lrp2*, respectively, while kidneys (positive controls) had high *LRP2/Lrp2* expression (Fig. S3A,B). In accordance, purified mouse splenocytes did not express *Lrp2* either (Fig. S3B). The only difference between the publicly available microarray data and our qRT-PCR results was the high abundance of *Lrp2* transcripts we found in mouse spleens, which might suggest expression of this gene by the stroma and/or non-immune parenchyma (Fig. S3B). These data suggested that megalin cannot be the exclusive R_SHBG_, and prompted us to search for other potential receptors using *in silico* tools. The potential SHBG interacting proteins, screened with *PrePPI* protein-protein interaction database and filtered for their localization in the plasma membrane of lymphocytes, are listed in Table 1. Expression levels of mRNAs of these proteins in lymphocytes were visualized on a heatmap, together with some lymphoid and female reproductive tissues (Fig. S3C).

**Table 1:** Cell surface receptor candidates for SHBG that are expressed by human leukocytes and have predicted and/or experimentally determined interaction with SHBG.

UniProt ID	Gene name	Protein name	Predicted Score	Experimental Score	Final Probability*
Q6UXB4	*CLEC4G*	C-type lectin domain family G member 4	12.8979	4625.64	0.990
P03372	*ESR1*	Estrogen receptor 1 (ERα)	21383.8		0.973
P15309	*ACPP*	Prostatic acid phosphatase		4625.64	0.885
Q8N6Q3	*CD177*	CD177 antigen		4625.64	0.885

The *PrePPI* protein-protein interaction database was used for *in silico* screening for SHBG binding partners. *Values ≥0.7 are listed.

### SHBG colocalizes with membrane estradiol receptor in B lymphocytes’ cell membrane

**Figure 5:**
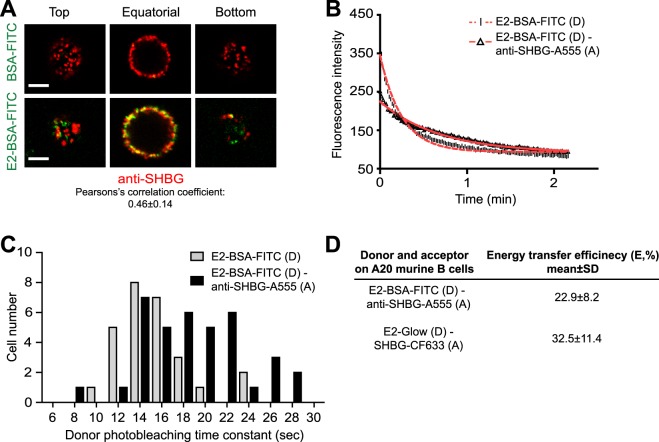
Surface-bound SHBG is in close proximity to membrane estrogen receptors (mERs) and E2 remains partly associated to SHBG during their internalization. (**A**) Representative confocal images show high colocalization (yellow) of E2-BSA-FITC (green: staining mER) and anti-SHBG (red: staining cell surface bound SHBG) on A20 mouse B cells, while BSA-FITC control did not bind to these cells. Pearson’s correlation coefficient was calculated from ≥100 ROIs/sample. Scale bar: 5 µm. (**B**) Bleaching curves of representative donor-labeled (D: E2-BSA-FITC, dashed line) and donor + acceptor-labeled (D + A: E2-BSA-FITC + anti-SHBG and anti-rabbit IgG-A555, continuous line) samples are displayed. The bleaching time constant (τ, the decrease of the initial fluorescence intensity value to its 1/e part) of the D + A-labeled sample is larger than that of the D-labeled sample. (**C**) FRET efficiency between D and A was calculated from the average photobleaching time constants, determined by confocal microscopic monitoring of donor-photobleaching kinetics in D-labeled and D + A-labeled cells, respectively, as displayed on the panel (≥50 ROIs/sample). (**D**) Calculated FRET efficiencies (E) between E2-BSA-FITC and anti-SHBG (A555) as well as fluorescent E2 (E2-Glow) and SHBG (SHBG-CF633) are shown. A555, Alexa Fluor 555; membrane estrogen receptor, mER; region of interest, ROI.

In order to study the relevance of the above findings for E2 uptake and signaling, we mapped the spatial relationship between the major cell surface binding sites for E2 and SHBG by confocal microscopy. A relatively high colocalization of cell surface SHBG and E2-BSA-FITC (labeling mERs) was found (colocalization index: 0.46 ± 0.14), while the negative control BSA-FITC did not bind to A20 B lymphocytes (Fig. 5A). These data indicated that SHBG and mER were located in a common compartment at the surface of these cells. The high FRET efficiency (22.9 ± 8.2%), calculated from photobleaching time constants of donor-only labeled (E2-BSA-FITC) and double labeled (E2-BSA-FITC donor and anti-SHBG/Alexa555 acceptor) samples, further confirmed this hypothesis (Fig. 5B–D). The relatively high FRET efficiency might mean that a large fraction of the labeled mERs and SHBGs were in close proximity, in good accordance with the colocalization data.

### SHBG promotes E2 internalization into lymphocytes

**Figure 6:**
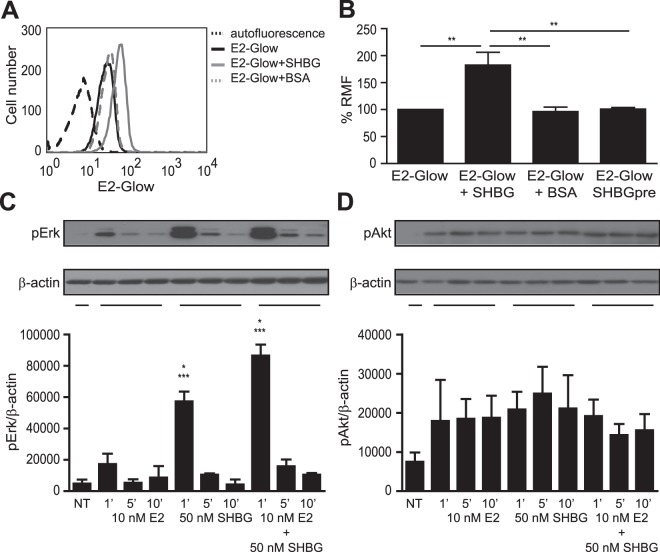
SHBG promotes E2 uptake by B lymphocytes and affects E2-induced rapid, non-genomic signaling. (**A**) A20 mouse B cells were incubated for 30 minutes with 10 nM E2-Glow previously mixed or not with 50 nM SHBG or BSA, and then fluorescence was detected by flow cytometry. Representative flow cytometric histograms are displayed. Dashed black line: autofluorescence; continuous black line: E2-Glow; continuous grey line: E2-Glow premixed with SHBG; dashed grey line: E2-Glow premixed with BSA. (**B**) The overall flow cytometric data on E2-Glow cellular uptake in the presence or absence of SHBG or BSA, or when cells pre-incubated with SHBG (SHBGpre), is represented as mean and standard error of mean (S.E.M.) values. RMF: ratio of the mean fluorescences of E2-Glow and autofluorescence. One-way ANOVA followed by Tukey’s post hoc test were used for the comparison of the groups (**P ≤ 0.01). Data are derived from at least three independent measurements. (**C** and **D**) A20 cells were left untreated or were stimulated for 1, 5 or 10 minutes with 10 nM E2, 50 nM SHBG, or E2 in complex with SHBG. Cell lysates were then subjected to immunoblotting with anti-pERK1/2 and anti-pAkt antibodies. Actin served as loading control. Representative cropped Western blot images from three independent experiments are shown together with densitometric quantification. Full-length blots are presented in Supplementary Fig. 6. Non-treated cells, NT. One-way ANOVA followed by Tukey’s post hoc test were used for the comparison of the groups (*P ≤ 0.05: SHBG 1’ vs SHB2 + E2 1; ***P ≤ 0.0001: SHBG 1’ or SHB2 + E2 1’ vs all other treatments). Data are derived from three independent measurements.

As a next step, we investigated whether SHBG can influence E2 uptake by incubating A20 B cells with fluorescent E2-Glow and then using flow cytometry and confocal microscopy for detection. The experimental system was first optimized without SHBG. Increasing the concentration of E2-Glow (Fig. S4A) or the incubation time at 37 °C (Fig. S4A,C) resulted in enhanced fluorescence of the cells, while the binding of E2-Glow at 4 °C was negligible. In addition, pre-treatment of cells with E2-BSA (binding to mERs), E2-Glow binding and uptake was reduced (Fig. S4B), suggesting that mER-dependent and -independent mechanisms are also involved in E2 transport across leukocyte plasma membranes. Furthermore, pre-complexing of E2-Glow with SHBG resulted in enhanced fluorescence of cells, while pre-complexing with BSA (Fig. 6A,B) or pre-incubation of cells with SHBG followed by the addition of E2-Glow (Fig. 6B) had no such effect. Of note, the proximity of E2 and SHBG was measured by FRET in the cytoplasm of A20 cells by incubating them with pre-complexed E2-Glow and SHBG-CF633. High FRET efficiency (32.5 ± 11.4%) was calculated (Fig. 5D), which indicated the molecular association of E2 with SHBG during the internalization process. These results suggest the functional involvement of SHBG in E2 uptake, which might influence the non-genomic E2 signaling.

### SHBG influences E2-mediated non-genomic signaling in lymphocytes

To explore the effects of SHBG in rapid E2 signaling, we treated A20 B cells with various combinations of SHBG and E2 and analyzed Erk1/2 and Akt activation by Western blot. SHBG induced a more robust phosphorylation of Erk1/2 than E2 did, with a peak at one minute (Figs 6C, S6). In addition, SHBG enhanced E2-induced Erk1/2 phosphorylation (Fig. 6C). E2, SHBG or E2-SHBG treatment at all time points resulted in Akt phosphorylation as well. In contrast to Erk1/2, no synergism could be observed between the two agents (Figs 6D and S6). These results indicated that upon binding to R_SHBG_, SHBG could activate MAPK/Erk and PI3K/Akt signaling pathways, and could also influence E2-mediated Erk1/2 signaling.

## Discussion

Several aspects of E2 transport mechanisms into cells of the immune system is still elusive, controversial or unanswered, therefore we aimed at providing a closer look into the possible contribution of sex hormone binding globulin (SHBG), a well-known transport protein for estrogens, in controlling the accessibility of E2 for immune cells.

Here, we found a considerable intrinsic *SHBG*/*Shbg* mRNA expression in T lymphocytes, but much lower or undetectable levels in human and mouse B cells, respectively. Furthermore, *Shbg* transcripts were present in mouse splenocytes and spleen, as well. These results are particularly interesting, since the local expression of SHBG may have autocrine/paracrine functions as suggested in prostate and granulosa-lutein cells^48,49^. In humans, the main source of SHBG is the liver, but its expression at the protein and/or mRNA levels have been found in other tissues including prostate, breast, and brain^50–52^. The apparent contradiction why we could not detect *Shbg* transcripts in livers of young, 6–8 weeks old mice by qRT-PCR, while publicly available RNA microarray data showed the presence of *Shbg* mRNA in postnatal livers with unknown age may be explained by the age-dependent expression of Shbg/*Shbg* in rodents^53^. Nevertheless, since the level of human *SHBG* in lymphocytes is low, liver-produced SHBG might be the primary source in humans, while other tissues in mice, that transports E2 to lymphocytes and the locally expressed SHBG may additionally fine-regulate this process in an autocrine/paracrine fashion.

In line with these, we found *in vitro* that external SHBG binds differentially to the surface of lymphoid cells. Mouse B lymphocytes bound the largest amount, while T cells did not bind detectable amounts of SHBG. Among human cells, primary B cells bound the largest amount, while primary T lymphocytes and T lymphoid cell lines bound less or no detectable SHBG, respectively. Results of our similar experiments using purified and fluorescently labeled SHBG further support this finding. The overall higher binding of purified and labeled SHBG to these cells over anti-SHBG binding may reflect the steric hindrance of surface bound SHBG eptiopes to the antibody used. Binding and internalization of SHBG into various cell types, such as prostate and breast cancer cells, hepatocytes and neurons have already been demonstrated by others^54^. Some groups utilized radioactive SHBG and plasma membrane isolates^55,56^, others used purified SHBG together with SHBG-specific antibody^6^ or fluorescent SHBG^45^ and intact cells to detect specific binding or internalization of SHBG into the cells mentioned above. All these data indicate that in contrast to the statement of the free hormone hypothesis^42^, SHBG may act not only as a passive carrier but also as an active transducer of estrogens through the plasma membrane.

Accordingly, it has been shown already that the putative receptor-binding domain of SHBG at residues 48–57 is almost perfectly conserved in all species studied^57^. Thus, it is not surprising that several research groups moved forward to the identification of R_SHBG_. Megalin (encoded by *LRP2*/*Lrp2*) was proposed as a putative R_SHBG_ in renal tubules and cancer cells^6,58^. However, we excluded megalin as an R_SHBG_ in lymphocytes, since we did not find herein mRNA expression of *LRP2* in these cells, in accordance with an earlier study showing the lack of immunoreactivity of human spleen and lymph node with a megalin-specific antibody^59^. In turn, based on our *in silico* screening, we propose additional receptor candidates expressed by lymphocytes, which may bind and internalize SHBG. ERα showed one of the highest interaction index (0.973) for SHBG, suggesting that it can also be a putative R_SHBG_. Detailed expression analyses of various ER forms confirmed the existence of the ERα46 splice variant on the cell surface of human leukocytes^17,60,61^. We earlier also confirmed the presence of ERα46 in cell membranes of mouse B lymphocytes by Western blotting plasma membrane isolates (unpublished data). Furthermore, an elegant study using EYFP and pHluorin-constructs of the ERα46 splice variant convincingly demonstrated that it is localized and functions as an integral transmembrane protein^24^. Of importance, Gnanasekar *et al*. suggested that R_SHBG_ is a multiprotein receptor complex^62^, which makes its exact identification more difficult. Nevertheless, validation of ERα46 or the other potential binding partners with high interaction index (C-type lectin domain family G member 4, CLEG4; Prostatic acid phosphatase, ACPP; CD177) as an R_SHBG_ needs to be performed in the near future.

Regarding the localization of R_SHBG_ in the plasma membrane of lymphocytes, we found that cell-bound SHBG is highly colocalized with lipid rafts^63–65^ as well as with mERs. The latter was also supported by FRET data indicating a close molecular proximity of R_SHBG_ to mERs. Association of mERs with lipid rafts has already been demonstrated by us, using impermeable E2-BSA ligand, further confirmed by the decreased binding of E2-BSA to lymphocytes with cholesterol-depleted membranes^30^. In accordance with this, ERα46 was found enriched in caveolar lipid rafts in MCF-7 breast cancer cells as well^66^. These results together further support our *in silico* finding that membrane-associated ERα46 may be an R_SHBG_. Of note, since lipid rafts are considered as important signaling platforms for cells, the association of R_SHBG_ with lipid rafts may indicate that SHBG can induce signaling through its receptor and then internalized in a raft-dependent way.

The question how SHBG guides the transport of sex steroids to/into target cells is highly controversial. Some reports proposed that only unliganded SHBG can bind to its receptor, which then binds sex steroids and mediates a subsequent signaling^67^. In contrast, other groups showed that ligands, pre-complexed with SHBG, are taken up by target cells^6,68^. Our present results on lymphocytes are concordant with the latter findings, as the uptake of fluorescent E2 was facilitated in B cells when it was pre-complexed with SHBG. Furthermore, the high FRET efficiency between fluorescent E2 and SHBG in the cytoplasm of these cells suggests that a significant fraction of E2 remains bound to SHBG during the internalization process. Indeed, it was already suggested that SHBG is internalized with the bound E2, and then intracellular organelles are responsible for shuttling E2 throughout the cells^58^. Taken together, the apparent contradiction concerning binding and uptake of E2-liganded SHBG is likely due to cell type-specific differences in this process.

In our experiments, the functional consequence of SHBG binding to cell membranes of lymphocytes was the activation of Erk and Akt pathways as shown by enhanced phosphorylation of these kinases. Furthermore, SHBG facilitated the uptake of E2 into lymphocytes resulting in enhanced Erk1/2 phosphorylation. The mediation of signaling by SHBG alone has also been shown by others using various cell types. For example, SHBG treatment led to Erk1/2 phosphorylation in HepG2 hepatocytes, which reduced levels of target mRNAs and proteins^69^. In addition, the incubation of cells with SHBG resulted in the accumulation of cyclic adenosine monophosphate (cAMP) as detected in cytotrophoblasts or in MCF-7 breast cancer cells, which could be further increased by the addition of steroid ligands^70,71^. E2-mediated non-genomic signaling *via* mER in leukocytes also involves the activation of these signaling pathways^72,73^. In accordance, we earlier found that E2-BSA treatment led to calcium response in mouse T cells, and also to the phosphorylation of Erk1/2 and Akt in T and B lymphocytes^22,30^. E2 alone did not induce proliferation of these cells; however, E2 decreased whereas E2-BSA enhanced activation-induced B and T cell proliferation^30^. All these results fit in the concept that estradiol and ERs exert either negative or positive regulatory effects on lymphocytes; this varies with type and activation state of cells, dose of E2, and expression levels of ERs or mERs^74^. Here, we propose that the SHBG/R_SHBG_ system further increase the complexity of this regulatory process.

Accordingly, genomic and non-genomic actions of E2 on lymphocytes may be altered during pregnancy, a condition characterized by elevated levels of SHBG (five- to six-fold increase) and E2 (approximately 50-fold increase) in the maternal serum^47^. It is widely accepted that lower physiological levels of E2 generally promote pathways leading to the production of various pro-inflammatory cytokines, while higher levels typically suppress immune response^74^. Nonetheless, a limited number of studies investigated how these changes contribute to the shift towards a Th2 type immune response and suppression of cell-mediated immunity during pregnancy, which results in a more tolerogenic state. The administration of E2 at similar concentrations as in pregnancy increased the number of regulatory T cells, reduced IL-17 production, and protected from experimental autoimmune encephalomyelitis, in an animal model of multiple sclerosis^75^. Another study showed that E2 dose-dependently enhanced IL-10 and IFNγ production of human T cells while its effect on TNFα production was biphasic, with enhancement occurring at low doses, and inhibition present at high concentrations^76^. Interestingly, here we found that the amount of cell surface-bound SHBG was lower in B lymphocytes from pregnant women than those from non-pregnant individuals, suggesting that the expression of the putative R_SHBG_ on the cell surface is tightly regulated according to the physiological state. Since circulating SHBG levels increase during pregnancy, reduced availability of R_SHBG_ might be responsible for the lower binding we observed. One supposed mechanism is downregulation of the R_SHBG_ gene expression, while others include an enhanced internalization/degradation (turnover) of R_SHBG_ protein in peripheral blood lymphocytes during pregnancy, similarly to the strong activation of T cell receptors^76^. This shift from R_SHBG_ signaling towards the free-hormone signaling in overall might contribute to the observed anti-inflammatory changes in lymphocytes during pregnancy. This important physiological phenomenon strongly warrants the investigation of the underlying complex mechanisms in details by later studies.

**Figure 7:**
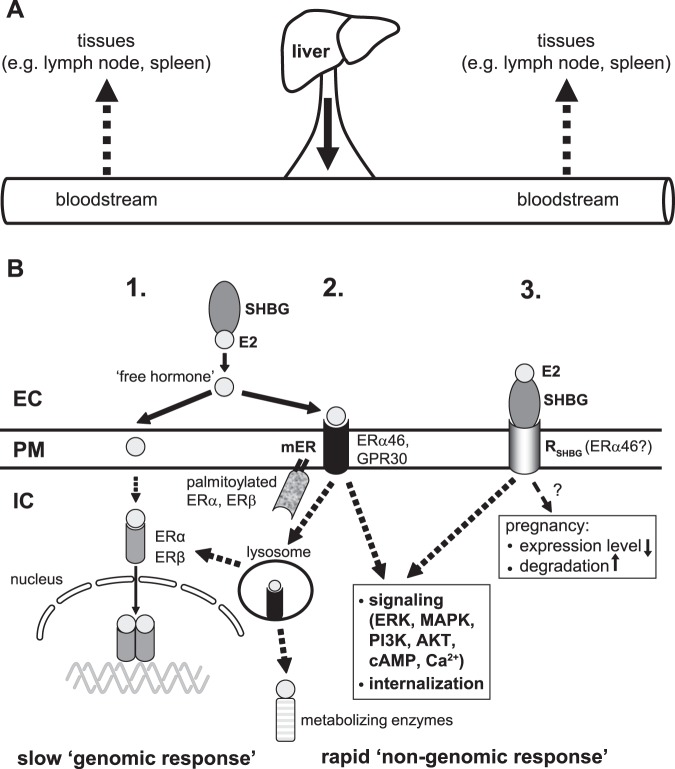
Schematic model for the possible E2 uptake pathways in leukocytes. (**A**) In human, SHBG is mainly produced by the liver than released into the bloodstream. From the bloodstream SHBG can be sequestered into various tissues, including lymphoid tissues. (**B**) According to the’free hormone hypothesis’, the transport of E2 through the plasma membrane takes place by passive diffusion. The classical cytoplasmic forms of ERα and ERβ – upon binding the hormone – act as inducers/regulators of transcription in the nucleus. (Pathway 1). E2, dissociated from SHBG, can also bind to a cell surface receptor (mER) and induce rapid signal transduction events such as increase of intracellular cAMP and calcium levels, activation of ERK, MAPK, PI3K or Akt pathways^30^. It can also be internalized and either used by metabolizing enzymes or act as a regulator of transcription through classical ERs. In the investigated immune cells either the truncated ERα46, as an integral membrane protein, or other palmitoylated, membrane-associated forms of ERs, and GPR30 may also function as mER (Pathway 2). SHBG with the bound E2 can bind to a membrane receptor and be internalized through an endocytic pathway. One possible candidate for this receptor (R_SHBG_) is ERα46, which is expressed in human lymphocytes and monocytes. In other cell types (such as neurons, fibroblasts, etc.), megalin (*LRP2*) may also function as R_SHBG_^84^ (Pathway 3).

In conclusion, our study provides novel insights on the importance of SHBG in E2-mediated regulation and the complexity of E2 actions in various cells of the immune system. Although still many questions remained unanswered, our results clearly show that cell surface binding and internalization of SHBG-E2 into lymphocytes may modulate E2-mediated signaling, highlighting a novel, SHBG/R_SHBG_-dependent entry pathway of E2 in these cells. Our model (Fig. 7) suggests that this novel pathway and the already described free and mER-mediated entry of E2 into target cells are not mutually exclusive. Cooperation of various mERs with each other and with classical ERs may result in a fine balancing of entry modes and subsequent immunomodulatory effects. This pathway may be altered in B lymphocytes in pregnant women that may be associated with changes in maternal immune homeostasis.

## Methods

### Cell lines, primary cells, and tissues

Human BL41 Burkitt’s lymphoma, and Jurkat E6.1 T leukemia cell lines were obtained from the European Collection of Authenticated Cell Cultures (ECACC, Salisbury, UK) and were cultured in RPMI 1640 medium. Mouse cell lines, IP12-7 T helper (Th) cell hybridoma^77^ and A20 B lymphoma (American Type Culture Collection; ATCC, Manassas, VI, USA) were cultured in RPMI 1640 (Merck-Sigma-Aldrich, St. Louis, MO, USA) medium as described earlier^78^. Media were supplemented with 10% fetal bovine serum (FBS), L-glutamine, Na-pyruvate, penicillin, and streptomycin (all from Merck-Sigma-Aldrich). To achieve E2-free conditions, cells were kept in phenol-red free medium supplemented with 10% charcoal-stripped FBS (Merck-Sigma-Aldrich) for 16–48 hours. To achieve SHBG-free conditions, cells were kept in FBS-free and phenol-red free RPMI 1640 medium for 16–24 hours.

Clinical sample and data collection were approved by the Health Science Board of Hungary (ETT-TUKEB 4834-0/2010-1018EKU). Informed consent was obtained from women prior to sample collection and the experiments conformed to the principles set out in the World Medical Association Declaration of Helsinki. Specimens and data were stored anonymously. Blood samples were obtained from five non-pregnant (pre-ovulatory phase) and 15 pregnant female healthy human donors, five-five each in the first, second and third trimesters of pregnancy. Peripheral blood mononuclear cells were isolated by Ficoll-Hypaque (Merck-Sigma-Aldrich) density-gradient centrifugation and washed in estrogen-free and SHBG-free medium before experiments.

Human kidney frozen biopsy was obtained from Prof. Jozsef Timar (2^nd^ Department of Pathology, Semmelweis University). Human liver frozen biopsy was provided by Prof. Laszlo Homolya (Molecular Cell Biology Research Group, Institute of Enzymology, Research Centre for Natural Sciences, Hungarian Academy of Sciences).

Adult C57BL6/J wild-type mice (Charles River, Budapest, Hungary) were maintained under a 12-h light/dark cycle at 23 °C, and they were supplied with water and food ad libitum. The breeding and the experiments were carried out according to the rules of the European Union-conforming Hungarian Act of Animal Care and Experimentation [1998, XXVIII, Section 243/1998, modified by 40/2013.(II.14)]. Sample collection was authorized by the Animal Health Directorate of the National Food Chain Safety Office (PEI/001/461-5/2013). Mice were sacrificed by cervical dislocation, and spleens, kidneys, and livers were removed, frozen immediately and kept at −80 °C until use. Splenocytes were also collected immediately after harvesting spleen tissues. Erythrocytes were lysed in ammonium chloride-Tris solution (pH 7.2). Splenocytes were used for flow cytometry or were frozen and kept also at −80 °C until use.

### RNA isolation, cDNA preparation and quantitative real-time RT-PCR

Cells and homogenized tissues were lysed in TRI reagent (Merck-Sigma-Aldrich) followed by total RNA isolation with the Direct-zol RNA MiniPrep kit (Zymo Research, Irvine, CA, USA) according to the manufacturers’ protocols. RNA concentrations were measured using the NanoDrop ND-1000 Spectrophotometer (Thermo Scientific, Wilmington, DE, USA), RNA integrity and quality were assessed by the Bioanalyzer 2100 (Agilent Technologies, Santa Clara, CA, USA). RNA samples were then stored at −80 °C until use.

The Maxima First Strand cDNA Synthesis Kit (ThermoFisher Scientific, Waltham, MA, USA) was used to generate cDNA. The reaction mixtures for qRT-PCR contained 20 ng of cDNA, a set of primers (Table S1) in 0.5 μM concentration, and the SYBR Green PCR master mix (ThermoFisher Scientific) in a total volume of 20 μl. Amplifications were run in 48-well optical reaction plates (ThermoFisher Scientific-Applied Biosystems, Foster City, CA, USA) using a StepOne amplification and detection system (ThermoFisher Scientific-Applied Biosystems) and Luminaris Color HiGreen ready-to-use solution (Thermo Scientific). Initial denaturations were performed at 95 °C for 10 minutes followed by 40 cycles consisting of denaturation at 95 °C for 15 seconds, and annealing and extension steps at 60 °C for 60 seconds. The cycle threshold (Ct) value of each reaction was obtained by the StepOne qRT-PCR analysis software (ThermoFisher Scientific-Applied Biosystems).

### *In silico* gene expression analysis

We used Genevestigator V6, a reference database of systematically annotated and high-quality microarray data from several organisms^79^, to find out how *SHBG* and *LRP2* genes are expressed in different tissues, primary cells and cell lines of interest in humans and mice. High-quality RNAseq data were obtained from the Genotype-Tissue Expression (GTEx) project^80^ for the analyses of the expression of *SHBG* and *LRP2* genes in human tissues and cells as well (https://www.gtexportal.org/home/).

### *In silico* SHBG-SHBG receptor interaction screening

The *PrePPI* (http://bhapp.c2b2.columbia.edu/PrePPI) database^81^, which combines predicted and experimentally determined protein-protein interactions (PPIs), was screened for potential SHBG receptors with default settings and probability values ≥0.7. The list of candidate SHBG interaction partners was filtered for cell surface proteins that are potentially expressed by leukocytes, using the UniProt (www.uniprot.org) and Expression Atlas (www.ebi.ac.uk/gxa/home) databases. Genevestigator-derived data was used for the generation of the heatmap that visualizes mRNA expression levels of these genes.

### Western blot

For the detection of SHBG at protein level, BL41, Jurkat, A20 and IP12-7 cells were pelleted and lysed in lysis buffer containing 20 mM Tris (pH 7.5), 150 mM NaCl, 1 mM EDTA, 1 mM EGTA, 1% Triton X-100, supplemented with protease inhibitors (1 mM phenyl methyl sulphonyl fluoride, 1.5 μM aprotinin, 10 μM pepstatin, 10 μM leupeptin; Merck-Sigma-Aldrich).

For the detection of Erk and Akt phosphorylation, A20 mouse B cells were treated with E2 (10 nM), SHBG (50 nM) or E2-SHBG mixture for 1, 5 or 10 minutes, and then were pelleted and frozen in liquid nitrogen. Pellets were lysed in lysis buffer described above, supplemented with 1 mM Na-orthovanadate phosphatase inhibitor (Merck-Sigma-Aldrich) as well.

In both cases, cell lysates were incubated with Laemmli buffer for 5 minutes at 95 °C, and then samples were subjected to 12% SDS polyacrylamide gelelectrophoresis (SDS-PAGE) followed by blotting to nitrocellulose membrane (Bio-Rad Laboratories, Hercules, CA, USA) with a semi-dry blotter (Bio-Rad Laboratories). Membranes were blocked with 5% BSA (in TBS with 0.05% Tween-20) for 1 hour, and then these were incubated overnight at 4 °C with primary antibodies (Table S2). After repeated washing with TBS-Tween buffer, blots were incubated with secondary HRPO-conjugated antibodies (SouthernBiotech, Birmingham, AL, USA) for 1 hour (Table S2). After repeated washing with TBS-Tween, protein bands were visualized by enhanced chemiluminescence (ECL; Amersham International, Little Chalfont, UK).Where needed, membranes were stripped and re-probed for loading control. Three independent experiment were done. Densitometry of Western blot bands was quantified by ImageJ software (https://imagej.nih.gov/ij/)^74^.

### Flow cytometry

To measure surface-bound SHBG, human and mouse lymphocyte cell lines were kept in RPMI supplemented with FBS (naturally containing bovine SHBG). In some cases, A20 cells were pre-incubated with 45nmol purified SHBG (Lee BioSolutions, Maryland Heights, MO, USA) in FBS-free RPMI, containing 0.3% BSA, for 30 minutes on ice before staining. Primary leukocytes of pregnant and non-pregnant women were transferred to ice immediately after isolation, in order to inhibit internalization of SHBG already bound to the surface of these cells in the blood. All subsequent steps were carried out in serum-free conditions. Fc receptors were blocked for 5 minutes, followed by incubation of cells with anti-SHBG to various species or rabbit IgG (isotype control) for 20 minutes on ice (Table S3). After washing, samples were incubated with goat anti-rabbit-IgG-Alexa Flour 488 (A488) secondary antibody for 20 minutes on ice, followed by repeated washing (Table S3). Primary cells were also stained with anti-human CD3-PE or anti-human CD19-APC (Table S3) to discriminate between T and B lymphocytes, respectively.

Binding of SHBG to B lymphocytes (human BL41 and mouse A20 cells) and freshly isolated primary leukocytes of pregnant and non-pregnant women was also measured by incubating them with purified SHBG (Table S3), conjugated with CF633 fluorophore (Mix-n-stain CF633 kit, Merck-Sigma-Aldrich). Incubation was carried out in PBS, containing 1% BSA, at 4 or 37 °C for 30 minutes, and then samples were washed with ice-cold PBS containing 1% BSA. Primary leukocytes were also stained with fluorophore-conjugated anti-human CD19-FITC or anti-human CD3-PE antibodies (Table S3). Inhibition of the binding of SHBG-CF633 was carried out by pre-treatment of cells with increasing amount of unlabeled SHBG at 37 °C for 15 minutes.

E2 uptake was monitored by incubating A20 B lymphocytes with the fluorescent E2-Glow (Table S3) in increasing concentrations or for increasing time periods in phenol-red free RPMI medium at 4 or 37 °C. In some cases, 10 nM E2-Glow was pre-mixed with 50 nM SHBG or BSA (1-hour incubation at room temperature), cells were pre-incubated with 0.5 mg/ml BSA or E2-BSA for 30 minutes at 4 °C or with 50 nM SHBG for 30 minutes at 37 °C in PBS followed by washing.

Flow cytofluorimetric measurements were carried out on a FACSCalibur device (BD Biosciences, San Jose, CA, USA) by collecting data from 10,000-50,000 cells. Data were analyzed using FCS Express V3 software (De Novo Software, Glendale, CA, USA). The relative mean of fluorescence (RMF) was calculated by dividing specific geometric mean fluorescence intensity with the geometric mean fluorescence intensity of the isotype control.

### Confocal laser scanning microscopy (CLSM)

To detect cell-membrane bound SHBG, mouse lymphocytes (A20, IP12-7) were stained with anti-SHBG as described above. Cholera toxin B (CTX-B)-A647 was applied to visualize plasma membrane. For intracellular staining, samples were fixed with 2% paraformaldehyde (PFA) for 15 minutes, washed, and then permeabilized with 0.1% saponin and 1% BSA in PBS for 10 minutes at room temperature. Cells were then incubated with anti-SHBG antibody applied in the permeabilization buffer for 20 minutes at room temperature. After washing, samples were incubated with goat anti-rabbit IgG-A488 secondary antibody for 20 minutes at room temperature in the same buffer and then washed.

Colocalization of mERs with SHBG was analyzed in the plasma membrane of A20 mouse B cells. Cells were labeled with the SHBG-specific antibody and then with goat anti-rabbit IgG-A555 secondary antibody as described above. Subsequently, cells were incubated with 1 mg/ml E2-BSA-FITC, a membrane-impermeable ligand of mERs, or 1 mg/ml BSA-FITC (as negative control) for 15 minutes at 37 °C, and then the unbound ligand was removed by washing.

For the visualization of SHBG binding and internalization, cells were incubated with SHBG-CF633 as described above. After fixation, plasma membranes were counterstained with CTX-B-A488 and nuclei with Hoechst 33342. For the measurements of fluorescence resonance energy transfer (FRET) between E2 and SHBG, cells were incubated with E2-Glow (donor only sample) or premixed E2-Glow and SHBG-CF633 (donor and acceptor sample) for 1 hour at 37 °C, and then the unbound complex was removed by washing. Cells were then fixed with 3.7% paraformaldehyde and were kept on ice until measurement.

Fluorescence microscopy was carried out on an Olympus IX81 inverted laser-scanning confocal microscope equipped with four lasers (UV: 405 nm; argon ion: 488 nm; He-Ne-green: 543 nm; and He-Ne-red: 632 nm excitation wavelengths) and FluoView500 software (Olympus Europe, Hamburg, Germany). Typically, fluorescence and DIC images (512 × 512 pixels) were acquired using a 60x oil-immersion objective (NA: 1.20). Images were processed using the ImageJ software (http://rsbweb.nih.gov/ij, National Institutes of Health, Bethesda, MD, USA). Colocalization was quantified by calculating Pearson correlation coefficients^65^ from at least 50 cells in each sample.

To confirm molecular proximity/interaction at the cell surface, FRET^82^ analyses between mER and SHBG molecules as well as between E2 and SHBG were carried out using the donor photobleaching technique, as described earlier^30^. The photobleaching decay curves (calculated from ≥50 ROIs) were fitted to exponential function in GraphPad Prism 5.0 (GraphPad Software, La Jolla, CA, USA) to get the photobleaching time constants (τ). FRET efficiencies (E) were calculated using the formula E = 1 − (τ*D*/τ*DA*)^83^.

### Statistical analysis

Statistical analysis was performed using GraphPad Prism 5.0. The One-way ANOVA test was used for the analysis of E2-Glow uptake and signaling studies. Unpaired t-test was used for the comparison of SHBG primary cell data obtained from non-pregnant and pregnant cases. Paired t-test was used for the comparison of anti-SHBG binding in the presence or absence of purified human SHBG. Results were considered statistically significant at *P ≤ 0.05 or **P ≤ 0.01.

## Supplementary information

Supplementary Information

## Data Availability

All data generated or analyzed during this study are included in this published article (and its Supplementary Information files).

## References

[1] Hess RA (1997). A role for oestrogens in the male reproductive system. Nature.

[2] Hewitt SC, Winuthayanon W, Korach KS (2016). What’s new in estrogen receptor action in the female reproductive tract. Journal of Molecular Endocrinology.

[3] Hammes A (2005). Role of endocytosis in cellular uptake of sex steroids. Cell.

[4] Villa A, Rizzi N, Vegeto E, Ciana P, Maggi A (2015). Estrogen accelerates the resolution of inflammation in macrophagic cells. Scientific Reports.

[5] Karpuzoglu E, Zouali M (2011). The multi-faceted influences of estrogen on lymphocytes: toward novel immuno-interventions strategies for autoimmunity management. Clinical Reviews in Allergy & Immunology.

[6] Dragin N (2017). Balance between Estrogens and Proinflammatory Cytokines Regulates Chemokine Production Involved in Thymic Germinal Center Formation. Scientific Reports.

[7] Blesson, C. S. Estrogen Receptors in Leukocytes - Possible Impact on Inflammatory Processes in the Female Reproductive System. In: Aimaretti, G (ed). Update on Mechanisms of Hormone Action - Focus on Metabolism, Growth and Reproduction. *InTech* pp 337–350 (2011).

[8] Gustafsson KL (2016). The role of membrane ERalpha signaling in bone and other major estrogen responsive tissues. Scientific Reports.

[9] Meyer MR (2014). G protein-coupled estrogen receptor protects from atherosclerosis. Scientific Reports.

[10] Almeida M, Han L, O’Brien CA, Kousteni S, Manolagas SC (2006). Classical genotropic versus kinase-initiated regulation of gene transcription by the estrogen receptor alpha. Endocrinology.

[11] Marino M, Galluzzo P, Ascenzi P (2006). Estrogen signaling multiple pathways to impact gene transcription. Current Genomics.

[12] Edwards DP (2005). Regulation of signal transduction pathways by estrogen and progesterone. Annual Review of Physiology.

[13] Carroll JS, Brown M (2006). Estrogen receptor target gene: an evolving concept. Molecular Endocrinology.

[14] Mendel CM (1989). The free hormone hypothesis: a physiologically based mathematical model. Endocrine Reviews.

[15] Fortunati N (1999). Sex hormone-binding globulin: not only a transport protein. What news is around the corner?. Journal of Endocrinological Investigation.

[16] Irsik DL, Carmines PK, Lane PH (2013). Classical estrogen receptors and ERalpha splice variants in the mouse. PloS One.

[17] Pierdominici M (2010). Estrogen receptor profiles in human peripheral blood lymphocytes. Immunology Letters.

[18] Hryb DJ (2002). Sex hormone-binding globulin in the human prostate is locally synthesized and may act as an autocrine/paracrine effector. The Journal of Biological Chemistry.

[19] Jensen EV, DeSombre ER (1973). Estrogen-receptor interaction. Science.

[20] Manolagas SC, O’Brien CA, Almeida M (2013). The role of estrogen and androgen receptors in bone health and disease. Nature Reviews Endocrinology.

[21] Mor G (2003). Interaction of the estrogen receptors with the Fas ligand promoter in human monocytes. Journal of Immunology.

[22] Adori M (2010). Estrogen augments the T cell-dependent but not the T-independent immune response. Cellular and Molecular Life Sciences.

[23] Soltysik K, Czekaj P (2013). Membrane estrogen receptors - is it an alternative way of estrogen action?. Journal of Physiology and Pharmacology.

[24] Kim KH, Toomre D, Bender JR (2011). Splice isoform estrogen receptors as integral transmembrane proteins. Molecular biology of the cell.

[25] Revankar CM, Cimino DF, Sklar LA, Arterburn JB, Prossnitz ER (2005). A transmembrane intracellular estrogen receptor mediates rapid cell signaling. Science.

[26] Yeung EH (2013). Adiposity and sex hormones across the menstrual cycle: the BioCycle Study. International Journal of Obesity.

[27] Liu L (2014). Estrogen inhibits LPS-induced IL-6 production in macrophages partially via the nongenomic pathway. Immunological Investigations.

[28] Benten WP, Lieberherr M, Giese G, Wunderlich F (1998). Estradiol binding to cell surface raises cytosolic free calcium in T cells. FEBS Letters.

[29] Guo Z, Krucken J, Benten WP, Wunderlich F (2002). Estradiol-induced nongenomic calcium signaling regulates genotropic signaling in macrophages. The Journal of Biological Chemistry.

[30] Schneider AE (2014). A dynamic network of estrogen receptors in mouse lymphocytes: fine-tuning the immune response. Journal of Leukocyte Biology.

[31] Falkenstein E, Tillmann HC, Christ M, Feuring M, Wehling M (2000). Multiple actions of steroid hormones–a focus on rapid, nongenomic effects. Pharmacological Reviews.

[32] Zeginiadou T, Kolias S, Kouretas D, Antonoglou O (1997). Nonlinear binding of sex steroids to albumin and sex hormone binding globulin. European Journal of Drug Metabolism and Pharmacokinetics.

[33] Hammond GL (2002). Access of reproductive steroids to target tissues. Obstetrics and Gynecology Clinics of North America.

[34] Pardridge WM (1988). Selective delivery of sex steroid hormones to tissues *in vivo* by albumin and by sex hormone-binding globulin. Annals of the New York Academy of Sciences.

[35] Selva DM, Hammond GL (2006). Human sex hormone-binding globulin is expressed in testicular germ cells and not in sertoli cells. Hormone and Metabolic Research.

[36] Pugeat M (2010). Sex hormone-binding globulin gene expression in the liver: drugs and the metabolic syndrome. Molecular and Cellular Endocrinology.

[37] Sun L (2012). Expression changes of sex hormone binding globulin in GDM placental tissues. Journal of Perinatal Medicine.

[38] Grishkovskaya I (2000). Crystal structure of human sex hormone-binding globulin: steroid transport by a laminin G-like domain. The EMBO Journal.

[39] Hammond GL (2011). Diverse roles for sex hormone-binding globulin in reproduction. Biology of Reproduction.

[40] Marquez DC, Chen HW, Curran EM, Welshons WV, Pietras RJ (2006). Estrogen receptors in membrane lipid rafts and signal transduction in breast cancer. Molecular and Cellular Endocrinology.

[41] Plymate SR (1991). Effects of sex hormone binding globulin (SHBG) on human prostatic carcinoma. The Journal of Steroid Biochemistry and Molecular Biology.

[42] O’Leary P, Boyne P, Flett P, Beilby J, James I (1991). Longitudinal assessment of changes in reproductive hormones during normal pregnancy. Clinical Chemistry.

[43] Caglar GS, Ozdemir ED, Cengiz SD, Demirtas S (2012). Sex-hormone-binding globulin early in pregnancy for the prediction of severe gestational diabetes mellitus and related complications. The Journal of Obstetrics and Gynaecology Research.

[44] Caldwell JD, Shapiro RA, Jirikowski GF, Suleman F (2007). Internalization of sex hormone-binding globulin into neurons and brain cells *in vitro* and *in vivo*. Neuroendocrinology.

[45] Cunningham M, Gilkeson G (2011). Estrogen receptors in immunity and autoimmunity. Clinical Reviews in Allergy & Immunology.

[46] Saade GR (2016). Development and validation of a spontaneous preterm delivery predictor in asymptomatic women. American Journal of Obstetrics and Gynecology.

[47] Lin AH (2013). Differential ligand binding affinities of human estrogen receptor-alpha isoforms. PloS One.

[48] Sinnecker G, Hiort O, Kwan PW, DeLellis RA (1990). Immunohistochemical localization of sex hormone-binding globulin in normal and neoplastic breast tissue. Hormone and Metabolic Research.

[49] Sinnecker G, Hiort O, Mitze M, Donn F, Neumann S (1988). Immunohistochemical detection of a sex hormone binding globulin like antigen in tissue sections of normal human prostate, benign prostatic hypertrophy and normal human endometrium. Steroids.

[50] Herbert Z (2005). Identification of sex hormone-binding globulin in the human hypothalamus. Neuroendocrinology.

[51] Li Y (2015). Age-dependent sex hormone-binding globulin expression in male rat. Ultrastructural Pathology.

[52] Hryb DJ, Khan MS, Rosner W (1985). Testosterone-estradiol-binding globulin binds to human prostatic cell membranes. Biochemical and Biophysical Research Communications.

[53] Nykjaer A, Willnow TE (2002). The low-density lipoprotein receptor gene family: a cellular Swiss army knife?. Trends in Cell Biology.

[54] Rosner W, Hryb DJ, Kahn SM, Nakhla AM, Romas NA (2010). Interactions of sex hormone-binding globulin with target cells. Molecular and Cellular Endocrinology.

[55] Krupenko NI, Avvakumov GV, Strel’chyonok OA (1990). Binding of human sex hormone-binding globulin-androgen complexes to the placental syncytiotrophoblast membrane. Biochemical and Biophysical Research Communications.

[56] Hammond GL (2016). Plasma steroid-binding proteins: primary gatekeepers of steroid hormone action. The Journal of Endocrinology.

[57] Hammond GL, Bocchinfuso WP (1995). Sex hormone-binding globulin/androgen-binding protein: steroid-binding and dimerization domains. The Journal of Steroid Biochemistry and Molecular Biology.

[58] Caldwell JD, Gebhart VM, Jirikowski GF (2016). Estradiol’s interesting life at the cell’s plasma membrane. Steroids.

[59] Saez-Lopez C (2017). Resveratrol Increases Hepatic SHBG Expression through Human Constitutive AndrostaneReceptor: a new Contribution to the French Paradox. Scientific Reports.

[60] Dunn JF, Nisula BC, Rodbard D (1981). Transport of steroid hormones: binding of 21 endogenous steroids to both testosterone-binding globulin and corticosteroid-binding globulin in human plasma. The Journal of Clinical Endocrinology and Metabolism.

[61] Madak-Erdogan Z, Lupien M, Stossi F, Brown M, Katzenellenbogen BS (2011). Genomic collaboration of estrogen receptor alpha and extracellular signal-regulated kinase 2 in regulating gene and proliferation programs. Molecular and Cellular Biology.

[62] Saez-Lopez C (2017). Sex Hormone-Binding Globulin Reduction in Metabolic Disorders May Play a Role in NAFLD Development. Endocrinology.

[63] Adams JS (2005). “Bound” to work: the free hormone hypothesis revisited. Cell.

[64] Consortium GT (2013). The Genotype-Tissue Expression (GTEx) project. Nature Genetics.

[65] Kiss E, Nagy P, Balogh A, Szollosi J, Matko J (2008). Cytometry of raft and caveola membrane microdomains: from flow and imaging techniques to high throughput screening assays. Cytometry Part A.

[66] Fortunati N (1999). Estradiol induction of cAMP in breast cancer cells is mediated by foetal calf serum (FCS) and sex hormone-binding globulin (SHBG). The Journal of Steroid Biochemistry and Molecular Biology.

[67] Keselman A, Fang X, White PB, Heller NM (2017). Estrogen Signaling Contributes to Sex Differences in Macrophage Polarization during Asthma. Journal of Immunology.

[68] Subramanian M, Shaha C (2009). Oestrogen modulates human macrophage apoptosis via differential signalling through oestrogen receptor-alpha and beta. Journal of Cellular and Molecular Medicine.

[69] Biro A (2007). Novel anti-cholesterol monoclonal immunoglobulin G antibodies as probes and potential modulators of membrane raft-dependent immune functions. Journal of Lipid Research.

[70] Lundgren S (1997). Tissue distribution of human gp330/megalin, a putative Ca(2+)-sensing protein. The Journal of Histochemistry and Cytochemistry.

[71] Gnanasekar M, Suleman FG, Ramaswamy K, Caldwell JD (2009). Identification of sex hormone binding globulin-interacting proteins in the brain using phage display screening. International Journal of Molecular Medicine.

[72] Hruz, T. *et al*. Genevestigator v3: a reference expression database for the meta-analysis of transcriptomes. *Advances in Bioinformatics 2008*, 420747 (2008). 10.1155/2008/420747 PMC2777001 19956698

[73] Monjas A, Alcover A, Alarcon B (2004). Engaged and bystander T cell receptors are down-modulated by different endocytotic pathways. The Journal of Biological Chemistry.

[74] Gallo-Oller G, Ordonez R, Dotor J (2018). A new background subtraction method for Western blot densitometry band quantification through image analysis software. Journal of Immunological Methods.

[75] Priyanka HP, Krishnan HC, Singh RV, Hima L, Thyagarajan S (2013). Estrogen modulates *in vitro* T cell responses in a concentration- and receptor-dependent manner: effects on intracellular molecular targets and antioxidant enzymes. Molecular Immunology.

[76] Gilmore W, Weiner LP, Correale J (1997). Effect of estradiol on cytokine secretion by proteolipid protein-specific T cell clones isolated from multiple sclerosis patients and normal control subjects. Journal of Immunology.

[77] Gogolak P (1996). Collaboration of TCR−, CD4− and CD28-mediated signalling in antigen-specific MHC class II-restricted T-cells. Immunology Letters.

[78] Gombos I, Detre C, Vamosi G, Matko J (2004). Rafting MHC-II domains in the APC (presynaptic) plasma membrane and the thresholds for T-cell activation and immunological synapse formation. Immunology Letters.

[79] Phiel KL, Henderson RA, Adelman SJ, Elloso MM (2005). Differential estrogen receptor gene expression in human peripheral blood mononuclear cell populations. Immunology Letters.

[80] Kovats S (2015). Estrogen receptors regulate innate immune cells and signaling pathways. Cellular Immunology.

[81] Taylor SE, Martin-Hirsch PL, Martin FL (2010). Oestrogen receptor splice variants in the pathogenesis of disease. Cancer Letters.

[82] Förster T (1948). Zwischenmolekulare Energiewanderung und Fluoreszenz. Annalen der Physik.

[83] Szentesi G (2005). Computer program for analyzing donor photobleaching FRET image series. Cytometry Part A.

[84] Wang C (2009). Oestrogen modulates experimental autoimmune encephalomyelitis and interleukin-17 production via programmed death 1. Immunology.

