# Supplementary material for: Sex hormone-binding globulin provides a novel entry pathway for estradiol and influences subsequent signaling in lymphocytes via membrane receptor
Source: Sci Rep. 2019 Jan 9;9:4. doi: 10.1038/s41598-018-36882-3 (PMC6327036; doi:10.1038/s41598-018-36882-3)
Supplement: Supplementary file 1 — Supplementary Information [file 41598_2018_36882_MOESM1_ESM.pdf]

# Sex hormone-binding globulin provides a novel entry pathway for estradiol and influences subsequent signaling in lymphocytes via membrane receptor

Andrea Balogh[1,2,#], Eva Karpati[1,2,#], Andrea E. Schneider[1], Szabolcs Hetey[2], Andras Szilagyi[3], Kata Juhasz[2], Gloria Laszlo[1], Petronella Hupuczi[4], Peter Zavodszky[3], Zoltan Papp[4], Janos Matko[1,*], Nandor Gabor Than[2,4,5,*]

[1]Department of Immunology, Eotvos Lorand University, Budapest, Hungary; [2]Systems Biology of Reproduction Lendulet Research Group, Institute of Enzymology, Research Centre for Natural Sciences, Hungarian Academy of Sciences, Budapest, Hungary; [3]Laboratory of Structural Biophysics, Institute of Enzymology, Research Centre for Natural Sciences, Hungarian Academy of Sciences, Budapest, Hungary; [4]Maternity Private Department, Kutvolgyi Clinical Block, Semmelweis University, Budapest, Hungary; [5]First Department of Pathology and Experimental Cancer Research, Semmelweis University, Budapest, Hungary

**[#]Contributed equally to the work**

***Correspondence:**
Nandor Gabor Than, MD, PhD
Systems Biology of Reproduction Lendulet Research Group, Institute of Enzymology, Research Centre for Natural Sciences, Hungarian Academy of Sciences
Magyar Tudosok krt 2.
H-1117 Budapest, Hungary
E-mail: than.gabor@ttk.mta.hu
ORCID ID: orcid.org/0000-0001-9385-7019

Prof. Janos Matko, PhD
Department of Immunology, ELTE Eotvos Lorand University
Pazmany Peter setany 1/C.
H-1117 Budapest, Hungary;
E-mail: janos.matko@ttk.elte.hu
ORCID ID: orcid.org/0000-0001-9434-934X

**Running title:** SHBG in estradiol uptake and non-genomic signaling in leukocytes

## Supplementary Table 1. Primer pairs used for qRT-PCR expression

| Gene | Species | Forward primer 5'-3' | Reverse primer 3'-5' |
|---|---|---|---|
| *SHBG* | human | gcccacgaccctccggct | cgtggtccagcacccacc |
| *Shbg* | mouse | tggacgatgggagatggc | tggcccagccagatatctc |
| *LRP2* | human | acatagttgctcatcactgttatcat | atgttgtatttggcgggttgatttc |
| *Lrp2* | mouse | agcagtttctctcgtcagag | ctcccaggtcctgcggttca |
| *ACTB/Actb* | human/mouse | ggctacagcttcaccaccac | gcgctcaggaggagcaatg |

## Supplementary Table 2. Western blot antibody dilution

| Antibody target | Clonality | Primary antibody dilution | Primary antibody source (Cat. #) | HRP-antibody (dilution) | HRP-antibody source (Cat. #) |
|---|---|---|---|---|---|
| SHBG | polyclonal | 1:200 | Santa Cruz Biotech. (sc-32890) | swine anti-rabbit IgG (1:4000) | DAKO (P0399) |
| pErk1/2 (Thr202/Tyr204) | polyclonal | 1:4000 | Cell Signaling Tech. (4370L) | swine anti-rabbit IgG (1:4000) | DAKO (P0399) |
| pAkt (Ser473) | polyclonal | 1:4000 | Cell Signaling Tech. (4060L) | swine anti-rabbit IgG (1:4000) | DAKO (P0399) |
| β-actin | monoclonal | 1:4000 | Merck-Sigma-Aldrich (A2228) | goat anti-mouse IgG (1:2500) | ThermoFisher Sci. (G-21040) |

## Supplementary Table 3. Flow cytometry and confocal microcopy antibodies/reagents

| Antibody/Reagent | Clonality | Source (Cat. #) |
|---|---|---|
| K9.361* anti-CD32b, mouse IgG2a | monoclonal | kind gift from Dr. Herbert C. Morse (NIH) |
| human FcR blocking reagent | - | Miltenyi Biotec (130-059-901) |
| anti-SHBG, rabbit IgG | polyclonal | Santa Cruz Biotech. (sc-32890) |
| rabbit IgG | polyclonal | Santa Cruz Biotech. (sc-3888) |
| goat anti-rabbit IgG-Alexa Fluor 488 | polyclonal | ThermoFisher Scientific (A-11008) |
| goat anti-rabbit IgG-Alexa Fluor 555 | polyclonal | ThermoFisher Scientific (A-21429) |
| anti-human CD3-PE, mouse IgG2a | monoclonal | Immunotools (21850034) |
| anti-human CD19-APC, mouse IgG1 | monoclonal | Immunotools (21270196) |
| anti-human CD19-FITC, mouse IgG1 | monoclonal | Immunotools (21270193) |
| SHBG-CF633 | - | Lee Biosolutions (527-30) |
| Estradiol Glow | - | Jena Biosience (PR-958S) |
| cholera toxin B-Alexa Fluor 647 | - | ThermoFisher Scientific (C34778) |
| cholera toxin B-Alexa Fluor 488 | - | ThermoFisher Scientific (C34775) |
| E2-BSA-FITC | - | Merck-Sigma-Aldrich (E6507) |
| BSA-FITC | - | ThermoFisher Scientific (A23015) |
| Hoechst33342 | - | ThermoFisher Scientific (62249) |

*****Ref.: Kimura S, Tada N, Nakayama E, Liu Y, Hammerling U. A new mouse cell-surface antigen (Ly-m20) controlled by a gene linked to Mls locus and defined by monoclonal antibodies. *Immunogenetics* 1981, **14**(1-2)**:** 3-14

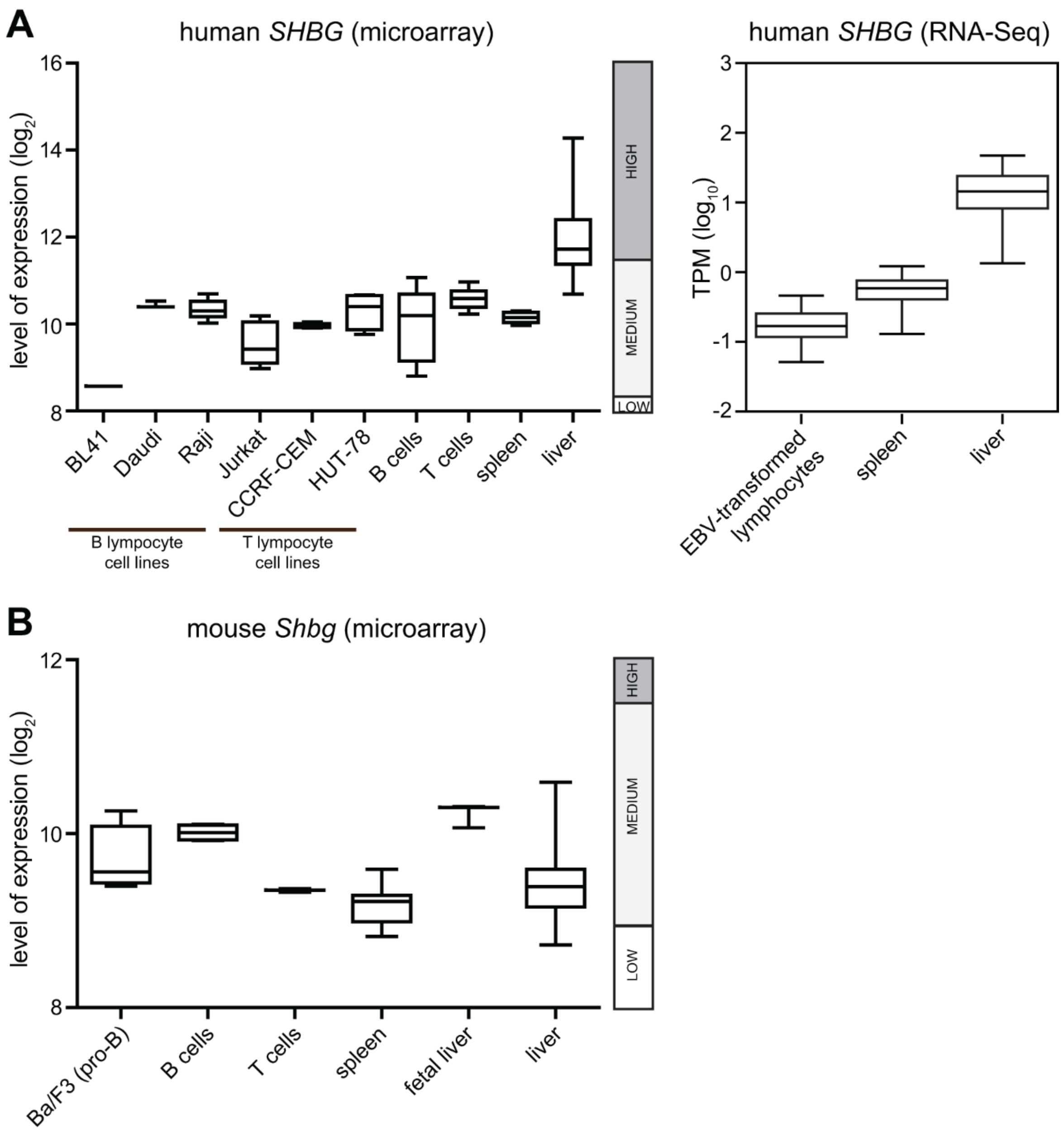


**Supplementary Figure 1. Microarray and RNA-Seq data show *SHBG*/*Shbg* expression in selected tissues and cells.** Box-plots (whiskers: min to max) represent *SHBG* mRNA expression levels in (**A**) human and (**B**) mouse selected lymphocyte populations, spleen, and liver. Data are derived from public databases (microarray: Genevestigator; RNA-Seq: GTEx project). Transcript per million, TPM.

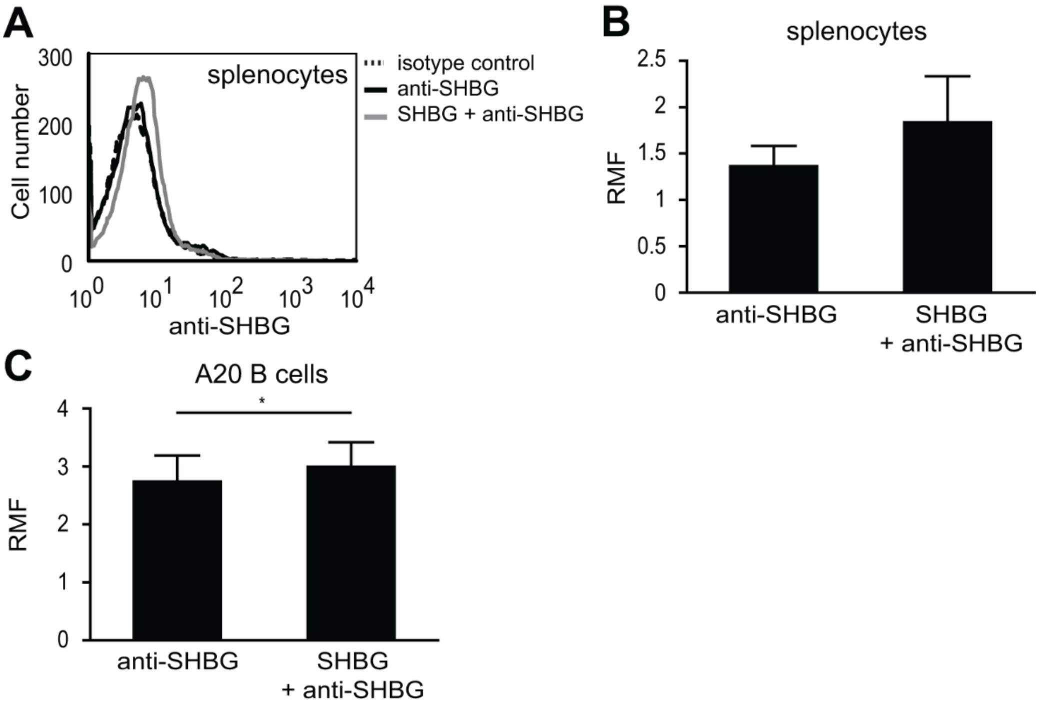


**Supplementary Figure 2. Binding of anti-SHBG to cells in the presence or absence of purified human SHBG.** (**A**) Mouse splenocytes were incubated or not for 30 minutes with 45nmol purified human SHBG and then cell surface-bound SHBG was detected by anti-SHBG antibody and A488-conjugated secondary antibody. Representative histograms are displayed. Continuous black line: anti-SHBG antibody; continuous grey line: purified SHBG + anti-SHBG antibody; dashed line: isotype control antibody. (**B** and **C**) The overall flow cytometric data on anti-SHBG binding to splenocytes or A20 B cells in the presence or absence of purified human SHBG is represented as mean and standard error of mean (S.E.M.) values. RMF: ratio of the mean fluorescence of anti-SHBG and isotype control antibody. Paired t-test was used for the comparison of the groups (*$P \leq 0.05$). Data are derived from three independent measurements.

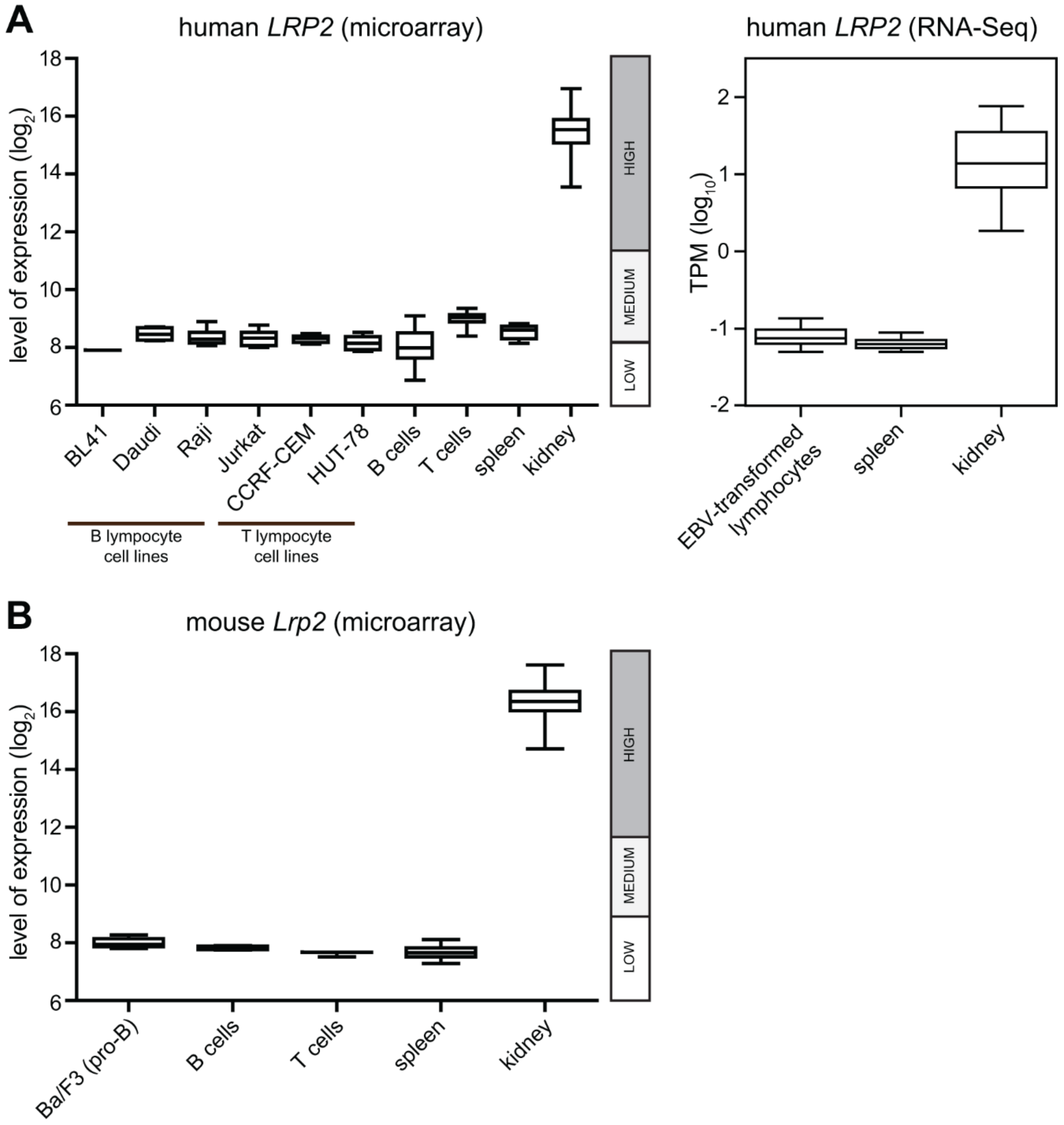


**Supplementary Figure 3. Microarray and RNA-Seq data show *LRP2*/*Lrp2* expression in selected tissues and cells.** Box-plots (whiskers: min to max) represent *LRP2*/*Lrp2* (encoding megalin) mRNA expression levels in (**A**) human and (**B**) mouse selected lymphocyte populations, spleen, and kidney. Data are derived from public databases (microarray: Genevestigator; RNA-Seq: GTEx project). Transcript per million, TPM.

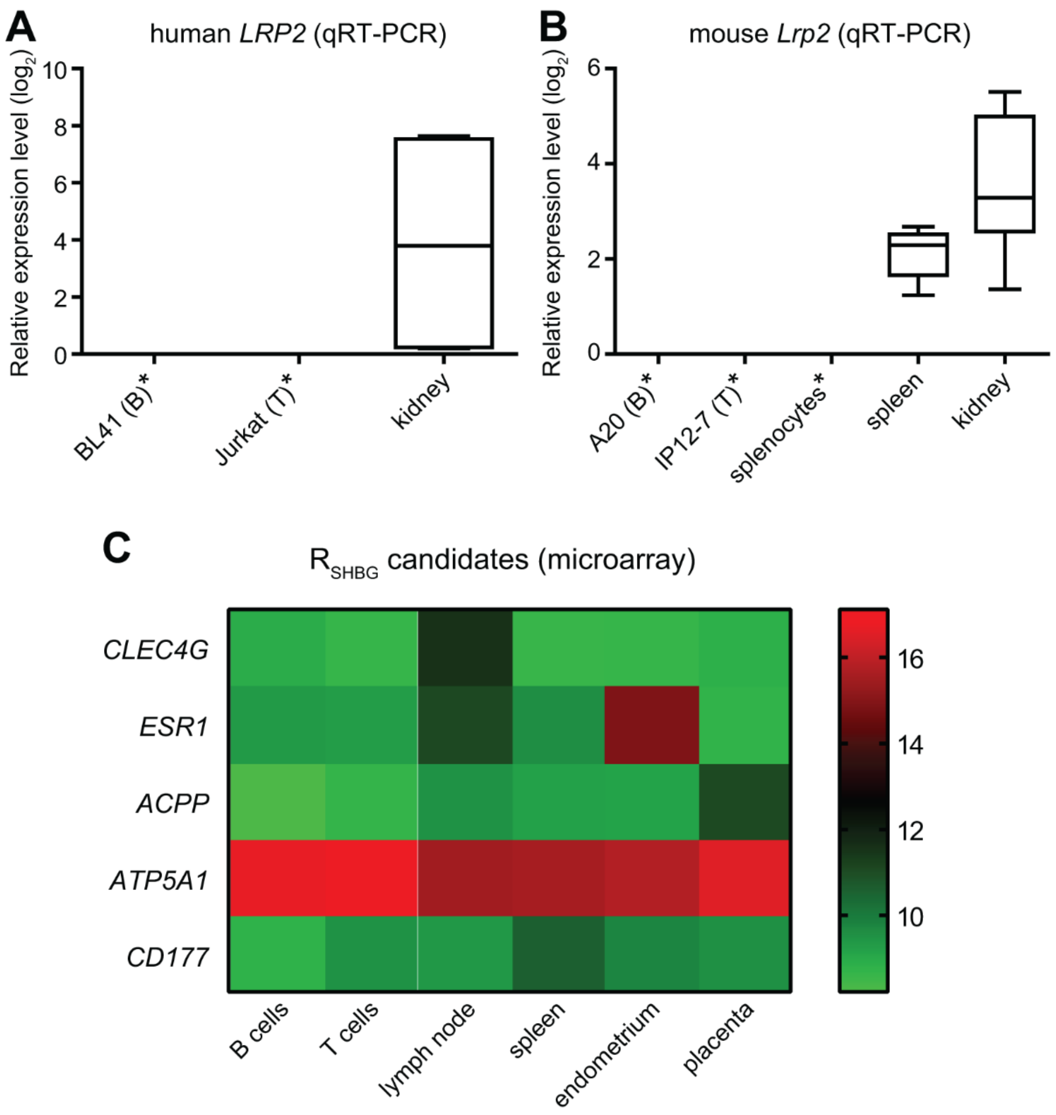


**Supplementary Figure 4. Expression of SHBG receptor candidates other than *LRP2/Lrp2* in selected tissues and cells.** (**A** and **B**) Box-plots (whiskers: min to max) represent *LRP2*/*Lrp2* (encoding megalin) mRNA expression levels, measured by qRT-PCR, in (**A**) human B cells (BL41) and T cells (Jurkat), as well as in (**B**) mouse B cells (A20), T cells (IP12-7), splenocytes and spleen. Kidney served as positive control in both human and mice. Relative expression level (-ΔCt with an arbitrary zero point) is denoted on the Y-axis. Three independent qRT-PCR experiments were performed in duplicates. Asterisks denote expression under the detection limit. (**C**) Heatmap represents mRNA expression levels of $R_{SHBG}$ candidates in human lymphocyte populations as well as in some lymphoid and female reproductive tissues. Data are derived from the Genevestigator public microarray database. Values are mean $log_2$ expression levels. Cluster of differentiation 177 antigen, CD177; C-type lectin domain family G member 4, CLEG4; Estrogen receptor 1, ESR1; Prostatic acid phosphatase, ACPP.

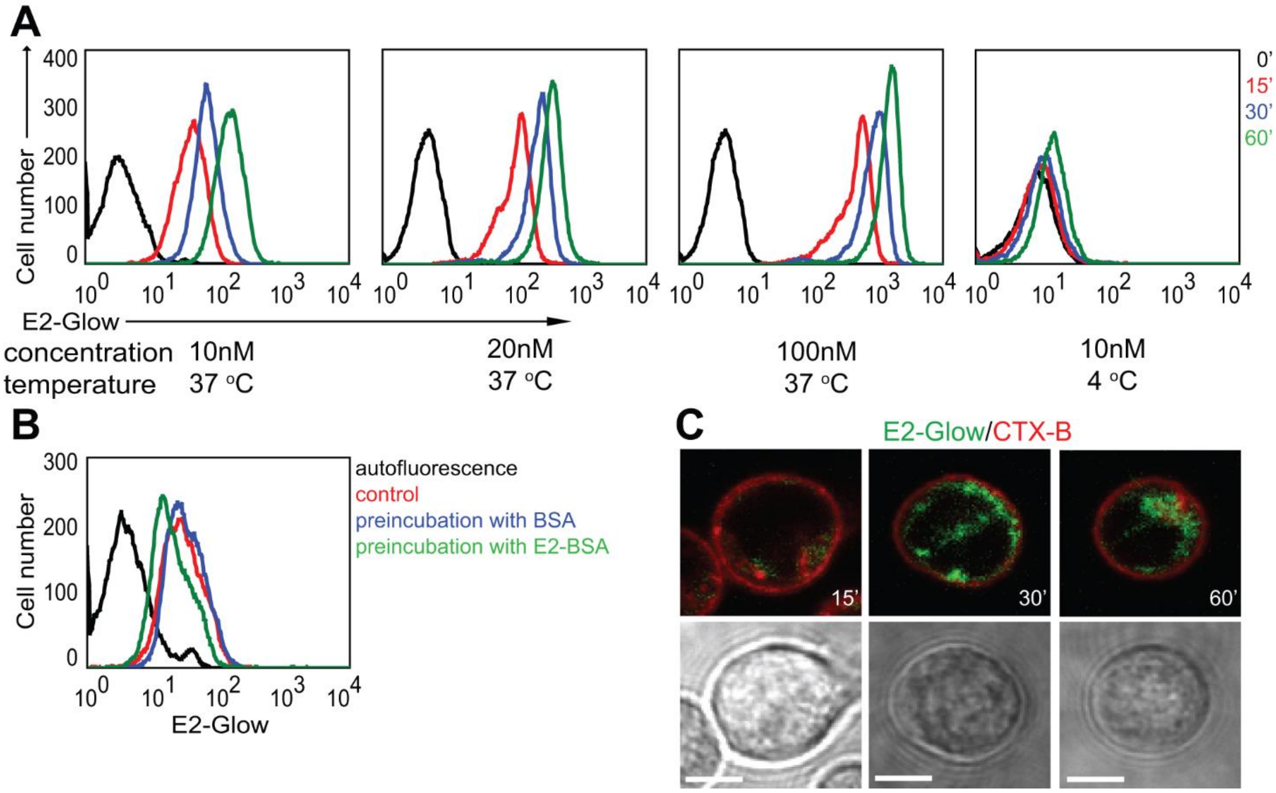


**Supplementary Figure 5. Characteristics of E2-Glow uptake by B lymphocytes.** (**A**) A20 mouse B cells were incubated with 0 nM (black), 10 nM, 20 nM, or 100 nM E2-Glow for 15 (red), 30 (blue), and 60 (green) minutes at 37 °C, or with 10 nM E2-Glow at 4 °C for the indicated times. Fluorescence was measured by flow cytometry. (**B**) Alternatively, cells were pre-incubated with E2-BSA (red) or BSA (blue) before addition of E2-Glow. Representative histograms are displayed. (**C**) Confocal images show an increasing intracellular signal of E2-Glow with increasing incubation time. Plasma membrane was counterstained with CTX-B-A647. Scale bar: 5 μM. Data are derived from three independent experiments. Cholera toxin B, CTX-B. A647, Alexa Fluor 647.

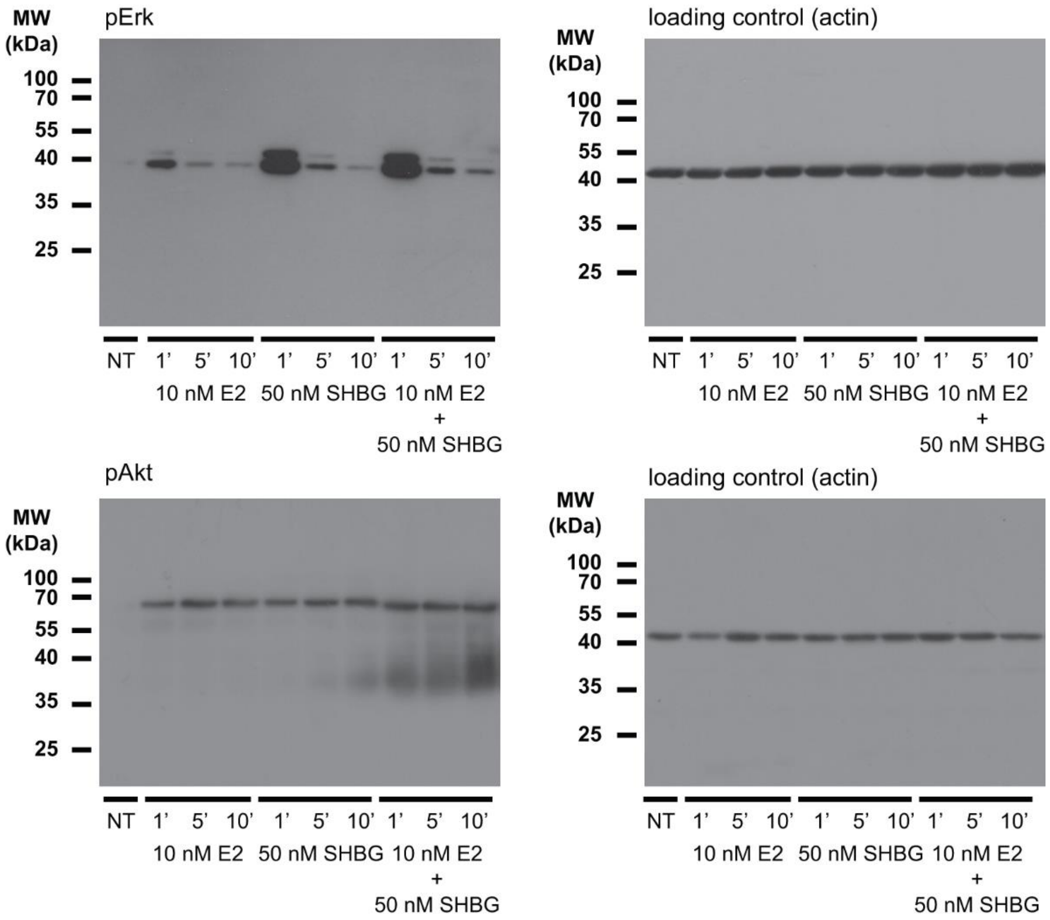


**Supplementary Figure 6. SHBG affects E2-induced rapid, non-genomic signaling.** A20 cells were left untreated or were stimulated for 1, 5 or 10 minutes with 10 nM E2, 50 nM SHBG, or E2 in complex with SHBG. Cell lysates were then subjected to immunoblotting. Two blots were run in parallel; one for anti-pERK1/2 the other for anti-pAkt antibodies. The blots were re-probed with anti-actin to ensure equal loading. Non-treated cells, NT.
